# Sweat Equity: Student Scholarships in Aotearoa New Zealand’s Universities

**DOI:** 10.1007/s40841-022-00244-5

**Published:** 2022-03-24

**Authors:** Max Soar, Lucy Stewart, Sylvia Nissen, Sereana Naepi, Tara McAllister

**Affiliations:** 1 Centre for Science in Society, Victoria University of Wellington, Wellington, New Zealand; 2 Te Pūnaha Matatini, University of Auckland, Auckland, New Zealand; 3 Toha Foundry Ltd, Wellington, New Zealand; 4 Department of Environmental Management, Lincoln University, Christchurch, New Zealand; 5 Department of Sociology, University of Auckland, Auckland, New Zealand

**Keywords:** Postgraduate, Scholarships, Inequity, PhD

## Abstract

This paper responds to calls from past and present students to increase the value of postgraduate scholarships in Aotearoa New Zealand. Here we provide context for understanding the scholarship landscape in Aotearoa, including how scholarships are understood in relation to dominant neoliberal framings of higher education and persistent inequities within the sector. We present data which provides insight into the current inequities in Summer, Masters and PhD scholarship values. The average value of PhD scholarships has remained stagnant between 2011 and 2019 resulting in the average being $11,238 less than the Living Wage in 2019. We show that the average length of time full-time PhD students take to complete their doctorates exceeds the three-year tenure of scholarships. We argue the status-quo of low scholarships, supplemented by postgraduate ‘sweat’, excludes people from participating in postgraduate education, preventing them and their communities from realising the public benefits that such an education can produce. We suggest that these inadequacies could be addressed through (1) raising Summer, Masters and PhD scholarships to the living wage; (2) extending tenure of PhD scholarships; and (3) reinstating the postgraduate student allowance.

**Supplementary Information:**

The online version contains supplementary material available at 10.1007/s40841-022-00244-5.

## Introduction

Increasingly there are calls from the research sector that postgraduate scholarships are insufficient for the increased cost of living in Aotearoa New Zealand. Through open letters (Morton, 2021), petitions (Hopkins, 2020; O’Connor & Hyde, 2021), surveys (Naepi, 2021; Simpson, 2021), and the news media (Guesgen, 2021; ‘Time for Leadership in Science’, 2021) researchers are consistently highlighting the inadequacy of scholarships and the urgent need to build stable career paths for emerging researchers. This paper provides a context for understanding the postgraduate research scholarship landscape in Aotearoa, including how postgraduate research scholarships are understood in relation to dominant neoliberal framings of higher education and persistent inequities within the sector.

The progression to postgraduate research studies is often presented as linear: a Summer scholarship and an Honours and/or research Masters progresses into a Doctor of Philosophy (PhD), which is configured as the ‘gateway’ into a research career. Each of these stages can be supported by scholarships. All eight universities in Aotearoa award summer scholarships, research Masters degrees, and PhDs. Summer scholarships are small, funded, and supervised projects that introduce scholars (usually undergraduates) to primary research over university holidays. Research Masters degrees often involve some combination of coursework and a significant self-directed research component and represent a first step into postgraduate research. PhDs in Aotearoa are similar in structure to those offered in the United Kingdom: supervised independent research towards the completion of a thesis, with coursework seldom required. All universities offer contestable scholarship support from internal funding, and some scholarships are also offered through external research funding schemes, including the Marsden Fund, the Endeavour Fund, or the National Science Challenges. Because the scholarships are contestable, not all postgraduate students receive scholarships. These scholarships typically encompass payment of university fees (if applicable) as well as a contribution to living costs and any other support, e.g. to attend conferences or pay for research costs. In this paper, we use the term ‘scholarship’ throughout, but our discussion is limited to the living costs portion (usually called the ‘stipend’).

While presented as linear, in practice the postgraduate research landscape is increasingly complex and fractured, and postgraduate student scholarships provide a pinch point that sits at the intersection of many persistent issues. Financial precarity among students and researchers has become an increasingly urgent issue over past decades, with the massification of higher education and increases in student debt (Cassuto, 2015), the expansion of the ‘gig economy’ within higher education (Acker & Haque, 2015; France et al. 2019), and rising costs of living (*Consumers Price Index: September 2021 Quarter*, 2021). Overlaying these developments are questions about the purpose and future of the research sector and workforce, including the potential for postgraduate research pathways to address persistent inequities within the sector (Naepi et al., 2017, 2019; Theodore et al., 2017). These challenges have been amplified with the Covid-19 pandemic, which has seen an acceleration of living costs as well as a growth in precarity in academic workforces (Nissen et al., 2020).

Within this complex terrain, we argue that there is a vital equity dimension that needs to be brought to the fore: current student scholarships have the potential to limit who can realistically complete a postgraduate research degree. This paper provides much-needed evidence that postgraduate student scholarships are inadequate: they do not reflect the increased living costs and are not aligned with Minimum Wage increases. Further, the length of scholarship awards often falls short of the median time it takes to complete a graduate degree. This data provides persuasive evidence of the urgent need to lift the value and length of scholarships in line with increasing living costs. While we focus on equity, it is important to acknowledge there may also be implications for recruitment. We make comparisons between scholarship payments and the minimum wage to demonstrate their insufficiency, but people considering postgraduate study will likely make their decision based on comparison with what they could earn in full-time employment on a graduate wage. For the purposes of this study, our focus is on domestic fees, while acknowledging there are significant and particular challenges affecting international students, with associated wellbeing implications.

In presenting this evidence, at times we consciously employ a language of ‘investment’, mirroring the logic used in academia to justify low scholarships, so we can demonstrate the shortcomings of this logic. We do so cautiously, recognising that such use risks further embedding and reinforcing neoliberal ways of thinking. As authors, we fundamentally oppose the neoliberal and managerial logics that structure, commodify, and marketise education, while being used as a justification to suppress wages. The title of this paper recognises the neoliberal understanding of education as a private good, a framing that has come to define much of the debate about postgraduate student scholarships. Much like a business, if you have no money you are expected to provide ‘sweat equity’—to invest time and energy through work, as part of a cost-sharing relationship, to earn any personal benefit or profit. The response to calls for increased scholarships so far can be understood in these terms: universities provide the qualification and in return postgraduates provide the sweat—they do the work. Students with fewer financial means must supplement their income, compromising academic performance, reducing extra-curricular opportunities, risking burnout, and embedding existing inequities. The status-quo of low scholarships, supplemented by postgraduate ‘sweat’, excludes people from participating in postgraduate education, preventing them and their communities from realising the public benefits that such an education can produce.

## Context

Scholarships sit at the intersection of multiple debates about the value of postgraduate research, which reflect wider shifts and contradictions within the higher education sector. It is clear, for example, that a PhD is no longer simply a mechanism for preparing one for a career in academia. The academic job market is saturated, leaving many early career researchers in fragmented and casual contracts for extended periods (Acker & Haque, 2017; Oldfield et al., 2021; Rao et al., 2021; Spina et al., 2020). In some cases, key research institutions have argued that there is a “mismatch between the relatively narrow skills” of PhD graduates and the “skills needed in employment roles” that would enable successful careers “of value to New Zealand” (Royal Society Te Apārangi, 2020, p. 22), implying that a PhD also inadequately prepares postgraduate researchers for a career *outside* academia. Yet postgraduate research is nevertheless valuable to universities and the wider research sector as research outputs, commoditised to stimulate the knowledge economy: the work of postgraduate researchers are included in productivity metrics like publications per million dollars spent (Ministry of Business, Innovation & Employment, 2021). Moreover, postgraduate research is increasingly positioned as an important site to address wider societal equity commitments, as highlighted in discussion documents like Te Apārangi Royal Society’s *Early Career Researchers in Aotearoa: Safeguarding and strengthening opportunity after COVID-19* (Nissen et al., 2020). Although often overlooked, postgraduate research can produce material benefits in and of itself—particularly when embedded in (and led by) communities and attuned to their needs (Halse & Mowbray, 2011).

Many of the tensions within the conceptualization of postgraduate research are rooted in neoliberal shifts in higher education in the 1980s. This period instigated a shift towards education as a commodity and individual investment, while maintaining some language about education as a public good. Alongside increasingly corporate management and branding of public services (Boston et al., 1996; Roper, 2018), structural shifts in university funding models saw universities shift away from exclusive, well-supported degree programmes where government funding kept tuition low and living allowances relatively universal, to a model that increased access by passing significant costs on to students (McLaughlin, 2003). The 1988 Hawke report advocated for a shift towards seeing higher education as a personal ‘investment’ with increased personal benefits which justified pushing costs onto individualised private citizens and away from the public purse (Hawke, 1988). To increase access to university without significantly increasing government spending, the Labour Government of the late 1980s abolished free fees for tertiary education, with the subsequent National Government of the 1990s introducing a student loan scheme and student allowances for students under 25 based on parental income (McLaughlin, 2003).

With these ‘cost-sharing’ reforms, postgraduate students saw increases in tuition fees, as well as an associated rise in student debt. These issues were brought to a head with the removal of the postgraduate student allowance in 2013 by the National-led Government. In justifying these reforms, the Ministry of Education noted the “value of postgraduate tertiary education” in terms of “social and economic benefits”, but also framed postgraduate study as a matter of individual investment, noting that “masters graduates earn more than bachelor graduates” and therefore “expected student loan repayment times are shorter on average for postgraduate borrowers than for other borrowers” (Education & Workforce Committee, 2019). Yet the logic of personal investment—that students pay for education in exchange for presumed subsequent benefits—obfuscates processes of ‘lean reproduction’ and exploitation that can underpin postgraduate study (Roper, 2018; Welch, 2015). Particularly for PhD researchers, the tertiary workforce is increasingly characterised by precarious short-term employment, competition with colleagues and unequal pay as university management attempts to produce ever more with less (Berg et al., 2016; Green et al., 2020). Student debt is also at unprecedented levels, with the removal of postgraduate allowances further amplifying reliance on student loans as well as other forms of debt (Nissen et al., 2019; Sin et al., 2018). While many Doctoral graduates move into a variety of jobs outside of academia, this transition is often fraught and involves extended periods of fragmented and precarious work, while also often repaying significant debt (Acker & Haque, 2017; Spina et al., 2020).

Studies have sought to explore the human costs of the financial conditions of graduate students. In a study of Otago University doctoral students during their first 2 years of candidature, Cornwall et al. (2019) identify how financial strain is interwoven with other stressors, which had a deleterious impact on students’ wellbeing and performance. Their research showed that many students juggled employment, in addition to their full-time studies, to meet their monthly financial obligations, regardless of whether they received a scholarship. These demands of employment together with the personal demands of family and care work left many students with time-related stress and guilt about allowing job demands to take time away from their study. Some students also connected their financial constraints to a sense of isolation, for instance making it difficult to see family. In addition, both international and domestic students expressed financial worries about maintaining personal finances once scholarship funding finished, especially given uncertainty about the PhD structure and timely completion (Cornwall et al., 2019). These pressures on doctoral students are likely to last throughout the rest of their candidature and may become especially amplified in the months before submission. Other studies of doctoral students internationally have identified that inadequate funding can be a hurdle to successful graduate student completion (Acker & Haque, 2015; Larivière, 2013; Yusuf et al., 2020).

There are vital equity issues embedded in questions of financial strain on postgraduate students. For instance, Acker and Haque (2015) remind us of the many students who do not have the benefit of “financial cushioning” to enable them to pursue their postgraduate study unencumbered by debt and worry. There are also potential flow-on effects. Inequity and exclusion are persistent issues within New Zealand’s research sector, despite commitments from the government and universities to value diversity and honour their obligations to equity under te Tiriti o Waitangi. Māori and Pacific scholars can experience systemic structural disadvantage and racism in universities, including the day-to-day reality of feeling isolated and the devaluing of Māori and Pacific knowledge, which can lead to the exclusion of Māori and Pacific bodies from universities (Kidman & Chu, 2019; McAllister et al., 2020; Naepi et al., 2019). Research by McAllister et al. (2019) and Naepi (2019) on the current numbers of Māori and Pacific academics show severe underrepresentation of Māori and Pacific scholars in New Zealand’s eight universities. Māori and Pacific comprise 16.5% and 7.5% respectively of the total population of Aotearoa, and yet comprise 4.8% and 1.7% respectively of academics. Naepi et al. (2019) have examined the period following a PhD for Māori and Pacific scholars. They identify a chronic under-representation of Māori and Pacific staff in both fixed-term contracts and permanent positions, suggesting that the pipeline into academia for Māori and Pacific scholars is fundamentally broken. Yet as the authors note, the idea of a pipeline alone is inadequate, and instead situate the experiences of PhD scholars within a wider context of navigating intersecting storms of racism, sexism, neoliberalism and neocolonialism, within the foundational whiteness of universities (Naepi et al., 2019, p. 153).

In New Zealand, fewer women enter the post-degree academic career pipeline than men and even once they do their work in academia is undervalued. Women, whose research career trajectories otherwise resemble men’s in terms of research performance, are promoted more slowly (Walker et al., 2020) and have smaller incomes, resulting in a lifetime gender pay gap of ~ NZ$400,000 (Brower & James, 2020). This means that the financial strain experienced by women as postgraduate students does not result in equitable outcomes in terms of academic opportunity, promotion, and pay. This situation is exacerbated globally by new managerialism in academia, which promotes precarity with gendered consequences (Steinþórsdóttir et al., 2019). There is no available data on the participation of people with disabilities in the New Zealand academy, but research in other countries shows that they are significantly underrepresented in postgraduate study, even compared to participation in undergraduate study (Booksh & Madsen, 2018). Academic spaces can be particularly contributors to poor mental health, with low pay and precarity being significant factors (Horton & Tucker, 2014). LGBTQIA + scholars, particularly transgender scholars, have also been shown to have more precarious and stressful existences in academic (and associated) spaces (Bonner-Thompson et al., 2021; Cech & Waidzunas, 2021). For all these groups, financial stress during postgraduate study represents an additional barrier to academic participation.

## Methods

There is currently a paucity of papers that explore inequities in scholarship values and how they have changed over time. Here, we have collated data about the value and tenure of scholarships from each of New Zealand’s eight universities. All scholarships are tax-free. We present data on the value and tenure of PhD, Masters and Summer scholarships. We ask three questions of the data:

1. how has the value of scholarships changed over time?
2. is there a mismatch between the time it takes to complete a PhD and the length of PhD scholarships?; and
3. how does the living cost contribution of the average doctoral scholarship compare to the Minimum Wage and the Living Wage?

A request for scholarship data was sent to each individual university in July 2021. Universities are not individually named in the data we present as we felt that anonymity would increase their willingness to contribute data. We requested three sets of data: (1) the value and duration of Summer, Masters and Doctoral scholarships from 2000 to 2020, 2) the length of scholarships and whether fees were included, and (3) the length of time to complete a PhD broken down by ethnicity and subject area. Doctoral scholarships and PhD completion data includes both Doctor of Philosophy and Doctor of Education. Our request was discussed at a Universities New Zealand (UNZ) meeting and UNZ then took it upon themselves to collate and provide the data request. UNZ acquired data for PhD completions through the IDI (Integrated Data Infrastructure). The IDI is a large, research database which houses de-identified data about people and households.^1^ Information on requirements attached to doctoral scholarships was obtained from University regulations published on their respective websites in October 2021 and presented in Table 2.^2^ Where restrictions on the amount of paid work were specified on a per annum basis in university documents, we divided by 52 weeks to calculate the average hours allowed per week.

**Fig. 1:**
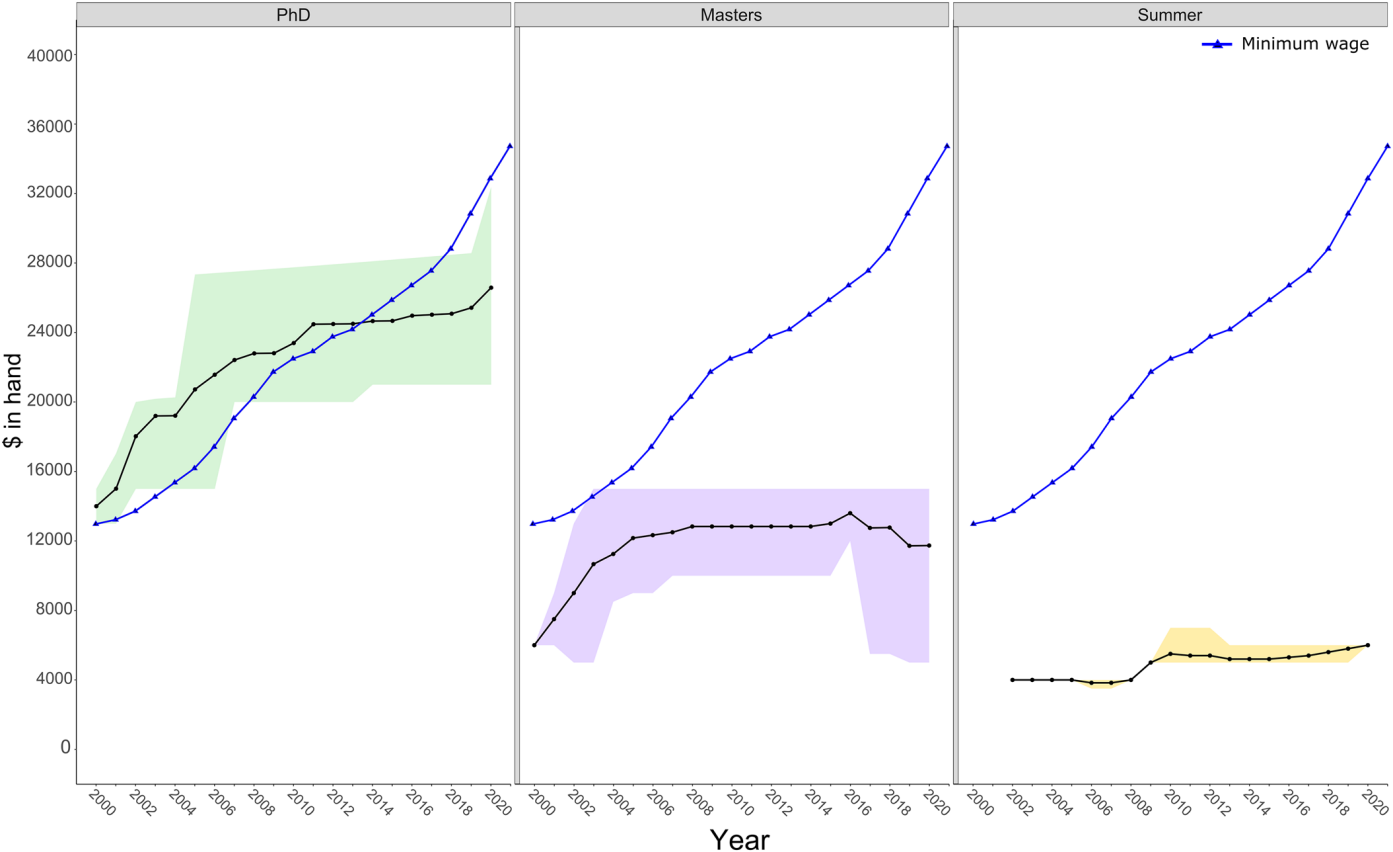
The average value (dollars in the hand) of Doctoral, Masters and Summer Scholarships at New Zealand’s eight Universities from 2000 to 2020. Shaded areas represent maximum and minimum values. Blue lines represent the Minimum Wage after tax

Some universities were unable to provide the full temporal data set we requested due to historical data not being recorded. Data for Summer scholarships was particularly patchy, with one university not providing any data despite offering Summer scholarships. Limited scholarship data was provided as a range of minimum and maximum values, rather than a single value. This variation and range are presented in Fig. 1, and we present averages only in Figure S1. Additionally, we are aware that individual researchers occasionally top-up PhD scholarships, our data does not account for this and it is unknown how widespread this practice is. PhD completion data obtained through the IDI was based on EFTS (Equivalent Full-Time Student) and excluded suspensions. The median EFTS provided in the data abstract for full-time students was then divided by 12 to calculate an approximate duration of PhDs.

## Results

### PhD Scholarships

The average PhD scholarship value, which are tax-free, increased steadily from 2000–2010, increasing from $14,000 to $24,476 respectively (Fig. 1). However, from 2011 to 2019, the average value of PhD scholarships remained stagnant. Over this period the highest and lowest scholarship values both changed by less than $1000. There was also large variation in scholarship values among universities (Fig. 1; Fig. S1). For example, in 2020, the largest PhD scholarship was $32,369 and the smallest was only $21,000 (Fig. S1). In 2014, the Minimum Wage (value after tax) exceeded the average value of PhD scholarships by $364 (Fig. 1) and this gap has continued to increase over time. In 2019, the average PhD scholarship was $5417 less than the Minimum Wage and $11,238 less than the Living Wage (Table 1).

**Table 1:** Annual living wage, minimum wage, and average PhD scholarship in 2019

Living wage	Minimum wage	Average PhD scholarship
$36,662	$30,841	$25,424

The wage figures are given after tax and ACC, to compare directly with the tax-free scholarships

All PhD scholarships are 3 years in length. At one university, however, recipients have, for the past 15 years, been able to apply for a six-month extension on their scholarship. Most PhD scholarships include paying tuition fees, on top of living costs. One university only began including fees in 2019 (data not shown).

### Masters Scholarships

Masters scholarships have persistently been below the Minimum Wage, with the exception of one university in 2003 only (Fig. 1). The average value of Masters scholarships increased from 2000 to 2008, but stagnated between 2008 to 2018. Interestingly, the average value of Masters scholarships has decreased in the past 5 years due to some universities introducing scholarships of lower values (Fig. 1). The maximum Masters scholarship value has remained at $15,000 from 2003 to 2020 (Fig. 1). The difference between the average Masters scholarship and the Minimum Wage in 2020 was $21,129.65 (Fig. 1). In 2020, the minimum Masters scholarship offered was $5500 (Fig. 1).

All Masters scholarships are one year in length. Two out of eight universities did not include fees in their scholarships. For a single year, one university offered Masters students with disabilities a scholarship with a two-year tenure, but subsequently ceased offering this arrangement (data not presented).

### Summer Scholarships

Although there is some variation among universities, the value of Summer scholarships has remained persistently low in the past 18 years (Figs. 1, S1). The average value varied from $4000 in 2002 to $6000 in 2020 (Fig. 1). Summer scholarships are usually for 10 weeks of full-time work.

### PhD Completions

**Fig. 2:**
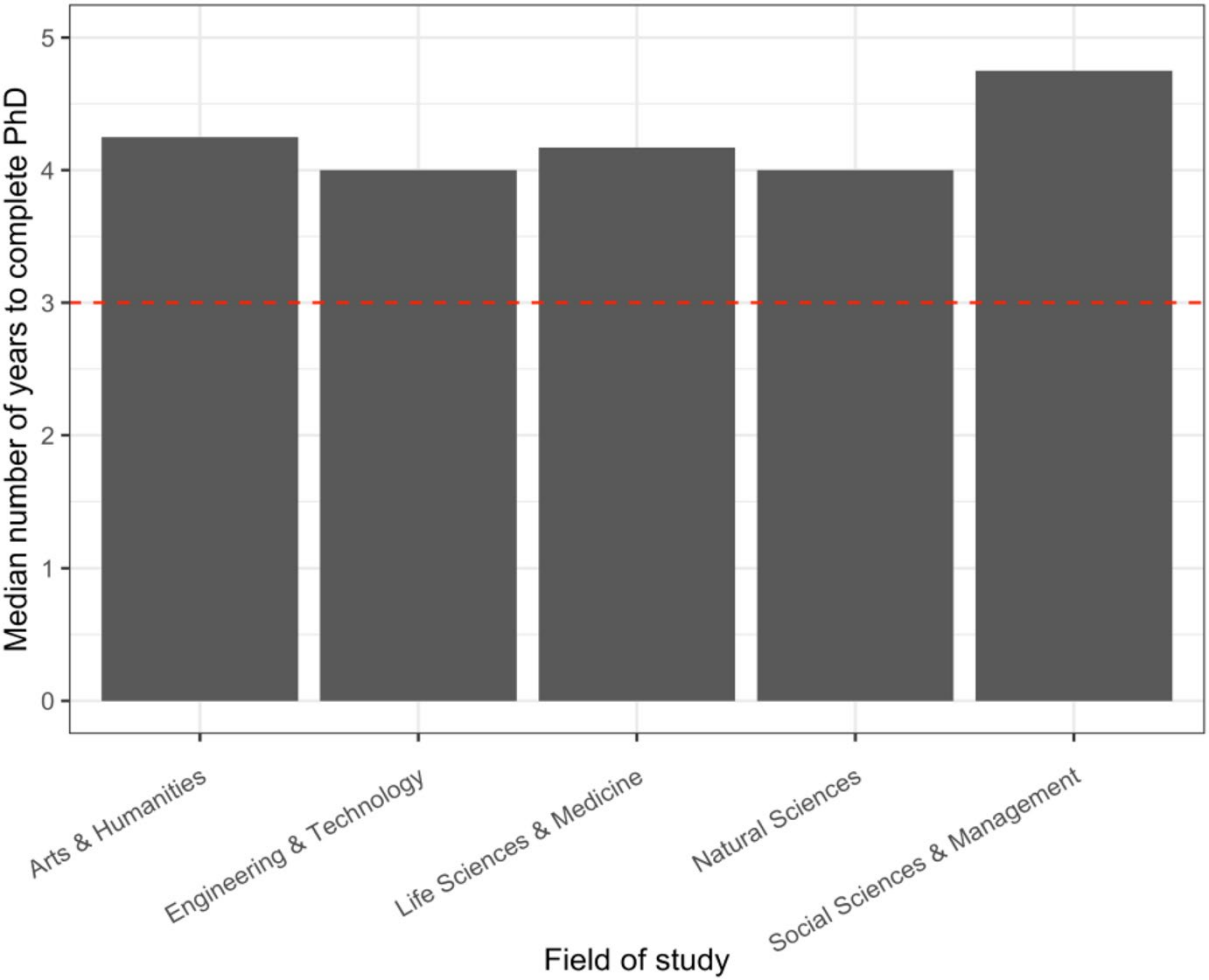
Median number of years for non-Māori to complete a PhD full-time from 2000 to 2020 by field of study

The median number of years to complete a PhD persistently exceeds the length of the three-year PhD scholarships regardless of the field study, by at least one year (Fig. 2). The median number of years was highest in the Social Sciences and Management at 4.75 years and lowest in Engineering and Technology and the Natural Sciences at 4 years (Fig. 2).

### Restrictions on Doctoral Scholarships

Only three of the eight universities in New Zealand include compulsory student service fees or levies in their doctoral scholarships. Doctoral researchers at the remaining five must pay compulsory annual fees ranging from $610.00 to $1017.60 (depending on the institution and campus; Table 2) to maintain full-time enrolment and access to their scholarship income.

**Table 2:** Scholarships included fees, extra costs to recipients, expected working hours, and restrictions on paid work for each university in 2021

University	Stipend	Tuition fees included	Student services Levy included	Expected hours worked p.w	Restrictions on paid work p.w.	Part-time arrangement
Auckland University of Technology	$25,000 p.a	Yes	Yes	At least 35 h	< 12 h	Unavailable
Lincoln University	$28,000 p.a	Yes	No: $850 p.a	Devote his or her whole time to study	< 10 h	Unavailable
Massey University	$25,000 p.a	Yes	Yes	40–50 h	< 10 h	$15,000 p.a. For ≤ 60 months
University of Auckland	$28,500 p.a. + annual cost of living adjustment	Yes	Yes	Full-time	< 10 h	Pro-rated
University of Canterbury	$21,000-$26,000 p.a	Yes	No: $892.80 p.a	Full-time	No information	Pro-rated
University of Otago	$27,000 p.a	Yes	No: $879 p.a	Full-time	< 10 h	Pro-rated
University of Waikato	$25,000 p.a	Yes	Yes	Full-time	< 10 h	Pro-rated
Victoria University of Wellington	$27,500 p.a	Yes	No: $1,017.60 p.a	At least 35 h	< 12 h	Pro-rated

Costs to recipients are given at the 2022 rates, except for the University of Otago for which the 2021 rates for the Dunedin Campus are the most up-to-date available (All data in Table 2 is taken from the most recent doctoral scholarship regulations published by each respective university on its website (AUT Doctoral Scholarship Regulations & Conditions, 2020; *Lincoln University Doctoral Scholarship Regulations*, n.d.; Massey University Doctoral Scholarship Regulations, 2021; *Regulations and Notes for the University of Otago Postgraduate Research Scholarships*, n.d.; *Scholarships and Awards—Massey University*, n.d.; *Scholarships: Lincoln University*, n.d.; University of Auckland Doctoral Scholarship Regulations, 2020; University of Canterbury Doctoral Scholarships Regulations, 2020; University of Waikato Doctoral Scholarship Regulations, 2021, n.d.; *Wellington Doctoral Scholarship Regulations|*Victoria University of Wellington, 2021). Full bibliographic details are included in References section.)

*p.a* per annum, *p.w* per week

Payment of doctoral scholarships are always contingent on full-time study, or pro-rated for part-time study where necessary. As such, recipients must agree to apply themselves to their studies full-time. Only three institutions specify an hourly expectation for full-time doctoral study. Victoria University of Wellington (VUW) and Auckland University of Technology (AUT) specify that a recipient must work on their research for at least 35 h per week (Table 2). Massey University specifies that students are expected to engage in 40–50 h of study per week (Table 2).

Many institutions allow recipients to take reasonable holiday leave on an annual basis without it affecting their entitlement. No New Zealand university, however, formally stipulates a recipient's entitlement to annual, parental, bereavement, or sick leave. Every university, except the University of Canterbury (UC), places explicit restrictions on the hours a recipient may work in addition to their research. Across these institutions scholars are limited to approximately 10–12 h of additional paid work per week, with some institutions specifying a weekly limit and others an annual limit of 500–600 h work within a calendar year. Such work is often subject to approval of a scholarship committee (Table 2).

Most universities place restrictions on co-tenure of their doctoral scholarships with other awards. In general, if a recipient receives other approved scholarships their university provided doctoral scholarship will begin to be attenuated once if it exceeds a specified amount. Most institutions allow a recipient to receive either an additional 30% or 50% of their scholarship value from another award before their entitlement is reduced. Massey allows co-tenure up to an additional $25,000; Lincoln caps recipient’s income from awards and scholarships at $35,000 total. None of the New Zealand university generic doctoral scholarships impose conditions on a doctoral researcher's intellectual property (IP)—this generally resides with the researcher.

## Discussion

Our findings have highlighted the inadequacies of research scholarships in Aotearoa. Not only is there a significant and growing gap between scholarships and Minimum/Living Wage, but they are also insufficient in terms of the time that it takes to complete a research degree. The neoliberal logics that currently guide our tertiary sector provide the justification for paying postgraduate researchers below-living wage. The cost-sharing approach to postgraduate research, where universities provide financial support while scholars provide sweat equity, is skewed in favour of universities, who have kept their financial contribution low relative to inflation and cost of living such that it is now incommensurate with the skilled labour of researchers. The understanding, by governments and universities, that tertiary education is both a public and private good, paid for in a cost-sharing relationship, hides the reality of living on a subsistence-scholarship that simultaneously restricts earning power through contractual limitations on hours worked. In this discussion, we consider the potential implications of this growing gap for developing a more inclusive and equitable research sector.

The gap between graduate scholarships and the cost of living raises concerns about who is able to engage in and complete postgraduate studies. There has been growing interest in developing supportive ‘pipelines’ into research to help address persistent inequities within the sector (e.g. Naepi et al., 2019). Disengagement from postgraduate studies is, however, evident in the decrease of progression onto postgraduate studies from 2006 to 2019, where the progression to a higher degree within 3 years from Honours/Postgraduate Diploma decreased from 24 to 18% and from 7 to 6% for Masters to a higher degree among domestic students (Rates of Progression to Further Study after Graduating, 2021). The number of students transitioning to postgraduate studies began to drop-off in 2014, a year after postgraduate living allowances were cancelled. The data on rates of progression to higher postgraduate degrees suggests that the government’s justification for continuing to delay the reinstatement of postgraduate allowances (because of total increases in enrolments) is faulty. These enrolments do not indicate an overall increase in the rate of participation. These concerns are especially apparent for Māori and Pacific scholars. While there are higher or similar rates of progression to graduate studies for Māori and Pacific communities, there are lower completion rates for Māori and Pacific students in bachelor or higher qualifications (Naepi et al., 2021). There is a lack of data on Masters and PhD scholars that would add the much needed in-depth understanding of how equity operates within the progression and completion rates, and how low scholarship values contribute to the ongoing under-serving of Māori and Pacific communities.

The gap between postgraduate scholarships and the cost of living also suggests a hidden toll for many of those who do engage in postgraduate research. With no allowance, the gap between scholarships and living costs needs to be met through other means. This resonates with other studies that have highlighted the juggling of multiple sources of financial support (Acker & Haque, 2015; Cornwall et al., 2019; Larivière, 2013). Those with the capacity to work in addition to their studies can make up some of the shortfalls; however, taking on the maximum allowed paid work (typically 10–12 h per week) in addition to full-time study results in a workload in excess 40 h per week which is nevertheless unlikely to pay commensurately with a living wage. Remaining shortfalls could be met through increased student loans (up to their caps), private debt and/or family contributions, if available. While there is a general increase in earnings for those who complete graduate degrees, these are field dependent (i.e. not all postgraduates benefit from increased earnings) and arguably not significant enough to justify high levels of debt or years of financial hardship. For instance, overall, the increase in earnings between a PhD and Masters 11 years after completing a degree is only $11,535 per annum, which arguably does not justify the increased debt taken on in fees and living costs. This costing does not take into account ethnic pay gaps which are shown to exist at a Bachelors degree or higher (Scott, 2021).

There is also the issue of the length of scholarships. The higher education sector values doctoral completion rates, as evident in the Research Degree component of the Tertiary Education Commission’s Performance-Based Research Funding (PBRF) which makes up 25% of the total yearly $321 million fund (Performance-Based Research Fund, 2021). This funding is calculated on a three-year rolling average of “research-based postgraduate degree completions, weighted by research volume, relative costs of the subject areas, and ethnicity and completions in Te Reo” (Performance-Based Research Fund, 2021). This creates a time-boundary for PhDs, as institutions can only claim up to 4 EFTS per doctorate per student, with any study exceeding that reported as “non-funded delivery” (The Tertiary Education Commission, 2021, p. 144). Yet these incentives to complete PhDs within 4 years are only met by Engineering and Technology and the Natural Sciences, indicating that there is a disconnect between the length of time it takes to complete a PhD and funding incentives. While scholarship deadlines of 3 years are frequently described as a ‘carrot’ to encourage students to complete, completions across all fields of study are consistently at least a year longer, leaving students to attempt to make up this financial shortfall. There is an important debate to have about the length of PhD stipends and the incentives and restrictions these provide for students and for supervisors. The concerns about equity we raise in this paper should be centred in these discussions. Although it has been beyond the scope of this paper, the disconnect between the length of a PhD and scholarships has particular implications for international students, who are also working within visa requirements.

Together, these shortfalls in scholarships mean that postgraduates who are not independently wealthy must accept sub-Minimum Wage incomes and a consequently reduced quality of life to engage in postgraduate study. The excessive workload necessary to meet the basic cost of living materially impacts the quality of research, as postgraduate students have less time to focus on their research, to rest, to think, and to engage in university life. It contributes to a culture of overwork and prevents working postgraduate students from accessing the (mostly unpaid) opportunities necessary to cultivate a research career. This requirement of overwork and underpayment systematically disadvantages those with disabilities, caring or familial responsibilities, and those without access to independent wealth who need a reliable, liveable income. Postgraduates who supplement their income through additional work may need to transition to part-time study to meet expenses, creating additional years of income lost compared to other work. The high cost of accommodation also means students could be limited, for extended periods of time, to cheaper, poor-quality accommodation, often involving significant commutes to city campuses, with associated demands on their time and potentially detrimental effects to their health and wellbeing. These disadvantages are exacerbated for Māori and Pacific scholars, who on average earn less than their Pākehā counterparts, are less likely to have access to familial wealth, and have been subject to colonial brutality and disempowerment (see Barber & Naepi, 2020; *Pacific Pay Gap Inquiry*, n.d.; Salesa, 2017; Schulze & Green, 2017). These systemic factors exclude people from participating in postgraduate education, preventing them (and their communities) from realising the public benefits that such an education can produce.

There are, however, relatively straightforward steps that could be taken to make a big difference for postgraduate students:

1. Raise Summer, Masters and PhD scholarships to match the Living Wage
2. Extend award tenure of PhD scholarships and national funding levers so scholarships reflect the actual time it takes to complete a postgraduate degree, including recognition of students with disabilities
3. Reinstitute the postgraduate living allowance

As a result of emerging researcher advocacy, some of these actions are already underway. A promising example in Aotearoa is the raising of scholarships by the Cancer Society to $40,000 per year, plus $10,000 per year in tuition fees, and a tikanga contribution of $10,000 across the course of the award (*Māori Cancer Researcher (Early Career) Awards 2021*, 2021). A more ambitious and comprehensive approach to addressing these issues could follow international precedent and treat PhD scholars as fixed-term employees for the duration of their studies. This would give scholars access to a wage that appropriately reflects their skill and expertise, leave allowances, professional development opportunities, and equal access to a union which could advocate for their working conditions (*Survey on the Structure of the Doctorates across Europe*, n.d.). This approach to scholarships could work well as part of wider discussions about more varied PhD programmes and industry linkages. Related options could involve restructuring scholarships to account for housing cost and location, childcare, and other cost of living factors, even extending to providing affordable housing close to campus. Such approaches would aim to improve the educational experience and create a less inequitable environment for post-graduates by providing an immersive environment where they live and work on campus, with realistic living costs accounted for, without the need for supplementary employment. Greater use of submission scholarships may also be an important mechanism by which to support students through an often financially precarious time.

By continuing to offer scholarships below Living Wage for highly skilled, home-grown researchers with the capability to work on world-leading research in Aotearoa, we devalue the knowledge that we develop and produce. At this point, it needs to be asked: who would—or is able to—sign up for a postgraduate degree with little income and low career prospects? It is increasingly urgent that we understand how universities have justified the levelling out of graduate scholarships so that we can challenge what is contributing to a decrease in graduate progression, inequity in our universities and growing frustration from graduate students about their ‘stolen wages’ (Cahill, 2021a,b; University of Sydney Casuals Network, 2020).

## Supplementary Information

Below is the link to the electronic supplementary material.

Supplementary file1 (DOCX 1563 kb)

## Footnotes

1 The results in Fig. 2 in this paper are not official statistics. They have been created for research purposes from the Integrated Data Infrastructure (IDI), managed by Statistics New Zealand.

2 As this paper was being prepared for submission, the University of Canterbury raised its PhD stipend to $28,000 p.a. plus tuition fees. This increase is not reflected in this paper and, while welcome, does not materially change the status quo or sufficiently address the inequity issues raised here.
