# Supplementary material for: Sweat Equity: Student Scholarships in Aotearoa New Zealand’s Universities
Source: N Z J Educ Stud. 2022 Mar 24;57(2):505–23. doi: 10.1007/s40841-022-00244-5 (PMC8943112; doi:10.1007/s40841-022-00244-5)

## Supplementary material

**Fig. S1** The value of PhD, Masters and Summer scholarship compared to Minimum Wage at each University from 2000 to 2020. Note: Summer scholarships are usually for a period of 10 weeks. University B and C provided a range for Masters scholarships and both values are plotted.


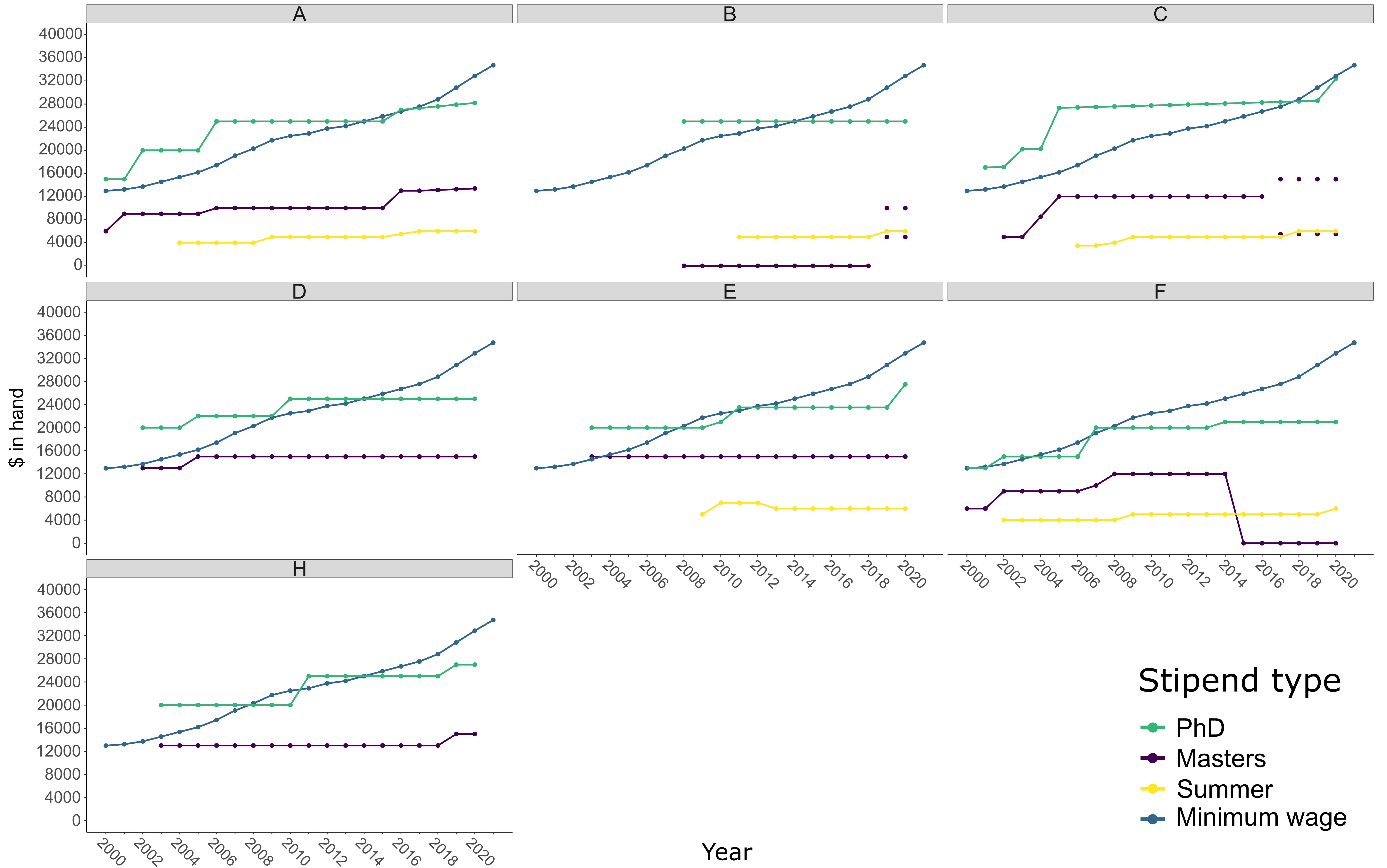

Supplement: Supplementary file 1 — Supplementary file1 (DOCX 1563 kb) [file 40841_2022_244_MOESM1_ESM.docx]
